# Evaluation of Pre-Treatment Assessment of Semaglutide Users: Balancing the Benefits of Weight Loss vs. Potential Health Consequences

**DOI:** 10.3390/healthcare13151827

**Published:** 2025-07-26

**Authors:** Faten F. Bin Dayel, Rakan J. Alanazi, Miteb A. Alenazi, Sahar Alkhalifah, Mohammed Alfaifi, Sultan Alghadeer, Abdulrahman Alwhaibi

**Affiliations:** 1 Department of Pharmacology, College of Pharmacy, Prince Sattam Bin Abdulaziz University, Al-Kharj 11942, Saudi Arabia; f.bindayel@psau.edu.sa; 2 Pharmacy Practice Department, College of Pharmacy, Alfaisal University, Riyadh 11533, Saudi Arabia; rjalanazi@alfaisal.edu; 3 Department of Pediatrics, College of Medicine, King Saud University, Riyadh 11451, Saudi Arabia; mitalanazi@ksu.edu.sa; 4 Pharmacy Department, Imam Muhammad Ibn Saud Islamic University, Riyadh 13317, Saudi Arabia; saalkhalifa@imamu.edu.sa; 5 College of Medicine, Alfaisal University, Riyadh 11533, Saudi Arabia; alfaifi911@gmail.com; 6 Department of Clinical Pharmacy, King Saud University, Riyadh 11451, Saudi Arabia; salghadeer@ksu.edu.sa

**Keywords:** semaglutide, Ozempic^®^, disease, history, laboratory, screening, assessment

## Abstract

**Background:** Although semaglutide (Ozempic^®^) is being prescribed off-label to individuals with obesity, some concerns have arisen regarding its use, particularly regarding the risk of thyroid and pancreatic disorders. Therefore, it is crucial to screen patients’ medical and family disease histories, as well as certain clinical parameters, before initiating this treatment for obesity or weight management. However, there is limited research investigating whether pretreatment assessment is adopted in clinical practice. **Method:** This is a single-center retrospective study involving adults who were prescribed semaglutide for obesity or weight management. Demographic data, comorbid conditions, semaglutide-related lab work, and disease history assessments, including pancreatitis, thyroid abnormalities, oculopathy, neuropathy, and any family history of thyroid cancer, were evaluated and documented prior to treatment initiation. **Results:** In total, 715 patients were included in the study, with an average age of 40.2 ± 12.0 years, and 49.5% of participants were male. The average weight and BMI prior to using semaglutide were 99.8 ± 18.1 kg and 36.3 ± 8.3 kg/m^2^, respectively, with predominantly overweight and obese individuals (collectively 91.3%). Approximately 69% of patients had 3–5 complications, with a high prevalence of cardiovascular and metabolic diseases before using semaglutide. Although HbA1c, serum creatinine, TSH, T3, T4, triglycerides, HDL, LDL, total cholesterol, and total bilirubin were monitored prior to semaglutide use, none of the patients’ pancreatic lipase, amylase, or calcitonin levels were measured. Although it is important to investigate all personal and family disease histories, including thyroid abnormalities, thyroid cancer, pancreatitis, retinopathy, eye problems, and neuropathy prior to semaglutide initiation, checks were only conducted in 1.8% of patients, despite 98.6% having at least one of the diseases assessed pretreatment. **Conclusions:** The current pretreatment assessment approach for patients prescribed semaglutide for weight reduction is underdeveloped, particularly with regard to assessing the influence of disease history on semaglutide use. This predisposes patients to a risk of severe clinical outcomes, including thyroid cancer, pancreatitis, and retinopathy.

## 1. Introduction

The milestone discovery of semaglutide, a glucagon-like peptide-1 receptor agonist (GLP-1RA), advanced the management of type 2 diabetes mellitus (T2DM) [1]. Given its ability to elevate insulin levels, inhibit glucagon secretion, and slow the digestive process via modulating the GLP-1 signaling pathway, semaglutide began to be manufactured by Novo Nordisk [2] and was approved first as a subcutaneous injection (Ozempic^®^) and then as an oral tablet (Rebylsus^®^) for glycemic control in adults with T2DM [3,4]. A significant reduction in major cardiovascular events was revealed, including cardiovascular death, nonfatal myocardial infarction, and nonfatal stroke, among T2DM patients in the later-performed SUSTAIN-6 trial, hence confirming the cardiovascular benefits of using Ozempic^®^ in these patients [5]. Besides its glycemic control and cardiovascular protection, semaglutide has recently gained momentum as a form of treatment for obesity, with notable outcomes in appetite regulation and weight loss. The global phase 3 Semaglutide Treatment Effect in People with Obesity (STEP) program (STEP trials) yielded remarkable evidence supporting its effectiveness in causing weight loss in diabetic and non-diabetic individuals; therefore, the FDA approved a higher dose of injectable subcutaneous formulations, marketed as Wegovy^®^ for chronic weight management in obese adults [6,7]. As such, given Ozempic’s^®^ significant effect on weight, it is commonly promoted and prescribed off-label for weight loss [8].

Similarly to many pharmacological interventions, semaglutide is associated with a spectrum of side effects, most commonly mild-to-moderate gastrointestinal symptoms such as nausea, vomiting, and diarrhea [9]. However, more serious adverse events have also been reported. Notably, there are concerns regarding the risk of acute pancreatitis [10] and thyroid disorders, including medullary thyroid carcinoma (MTC) [11]. While MTC cases have been reported with GLP-1 receptor agonists such as liraglutide, preclinical animal studies involving semaglutide have demonstrated an increased incidence of C-cell tumors in rodents [12]. Furthermore, some observational studies reported an increased risk of pancreatitis in association with GLP-1 receptor agonists, which has raised concerns about the overall safety of this treatment [13].

A critical assessment of the patient’s medical history and certain clinical parameters influenced by semaglutide is crucial before initiating this treatment for obesity or weight management. The AACE/ACE (American Association of Clinical Endocrinologists/American College of Endocrinology) obesity guidelines state that extreme caution is required before starting semaglutide if a history of pancreatitis, gallbladder disease, or severe renal impairment with eGFR < 30 mL/min is present [14]. In addition, patients with a history of MTC or multiple endocrine neoplasia syndrome type 2 (MEN 2) were found to be ineligible for treatment. In the SUSTAIN-1 trial, pretreatment screening for HbA1c, fasting plasma glucose, renal function, pancreatic and thyroid issues, blood pressure, and heart rate was conducted before semaglutide initiation [3]. More importantly, patients were excluded from the study if they had type 1 diabetes mellitus (T1DM), a history of diabetic ketoacidosis (DKA), severe hypoglycemia episodes, thyroid cancer risk, a personal or family history of MTC or MEN 2, chronic or acute pancreatitis, severe renal impairment with eGFR < 30 mL/min, NYHA class IV heart failure, or recent CV events within 3 months (e.g., myocardial infarction (MI) or stroke). In the SUSTAIN-6 trial, there was a statistically significant 76% increase in the risk of diabetic retinopathy complications (DRCs) with semaglutide use compared to a placebo [15]. Notably, patients experiencing rapid HbA1c reductions (>1.5–2% within 3–6 months) had higher rates of diabetic retinopathy (DR) progression. Interestingly, most DR events occurred in patients with pre-existing DR. Therefore, these patients, alongside those with high-risk features, should undergo a comprehensive eye exam before starting semaglutide. In the STEP 1 trial, individuals with a history of malignant neoplasms within the past 5 years, a medical history of MEN2 or MTC, elevated plasma levels of calcitonin, renal impairment with eGFR < 15 mL/min/1.73 m^2^, a history of major depressive disorder, psychiatric disorders (e.g., schizophrenia and bipolar disorder), or a history of suicide attempts were not eligible for treatment [7]. Additionally, individuals with calcitonin ≥ 100 ng/L, thyroid-stimulating hormone (TSH) > 6.0 mIU/L or <0.4 mIU/L, or chronic pancreatitis were excluded. In the STEP 2 trial, the importance of thorough pretreatment screening before initiating semaglutide was reinforced, particularly for renal function and the risk of pancreatitis and thyroid cancer [6]. Overall, all aforementioned trials reiterated the importance of pretreatment screening for certain diseases and laboratory testing prior to semaglutide use since it may cause or worsen such conditions.

Given the widespread use of semaglutide among patients with various medical histories and the potentially elevated risk of serious adverse events in certain patient populations due to personal or their family medical history, a critical evaluation of the suitability and safety of semaglutide for these patients is crucial before initiating obesity or weight management. The overall goal of this study is to determine the pretreatment assessment of a patient’s medical history and certain clinical parameters prior to initiating Ozempic^®^ for obesity or weight management, particularly those that influence or aggravate the risk of serious adverse events occurring.

## 2. Method

### 2.1. Study Design and Setting

This is a retrospective, non-interventional study involving patients who were prescribed semaglutide (Ozempic^®^) during outpatient clinic visits at King Khalid University Hospital, affiliated with King Saud University in Riyadh, Saudi Arabia, between December 2021 and December 2024. Following institutional review board (IRB) approval, patient recruitment was conducted by extracting data from hospital medical records. Participants were included if they were adults (aged ≥18 years) and prescribed semaglutide. Individuals were excluded if demographic data or medical history were missing.

### 2.2. Data Collection

A standardized data collection form was developed to extract variables relevant to the study’s objectives from the electronic medical records of patients. This included demographic data (such as age and gender), anthropometric measures (height, weight, and BMI), and comorbid conditions (e.g., dyslipidemia, hypertension, stroke, and cardiovascular diseases). The baseline laboratory results prior to treatment initiation were recorded, including the levels of glycosylated hemoglobin (HbA1c), serum creatinine (Scr), TSH, Triiodothyronine (T3), thyroxine (T4), triglycerides (TGs), HDL (high-density lipoprotein), LDL (low-density lipoprotein), total cholesterol, and total bilirubin. Medication details were also documented, including initiation date and its indications. Additionally, it was also assessed whether disease history, including pancreatitis, thyroid abnormality, ocular and neuropathic pathologies, and any family history of thyroid cancer, were evaluated prior to treatment initiation.

### 2.3. Statistical Analysis

All statistical analyses were performed using SPSS software version 28 (IBM Corp., Armonk, NY, USA). Descriptive statistics summarized patient characteristics, clinical measurements, and comorbidities. Continuous variables are presented as means with standard deviations (means ± SDs), while categorical variables are reported as counts and percentages (n, %). As the study aims were descriptive, no inferential statistical testing was conducted.

## 3. Results

### 3.1. Baseline Characteristics

A total of 715 patients were included in this study. The baseline characteristics table (Table 1) presents the demographic and clinical features of the study population. The average age of participants was approximately 40.2 ± 12.0 years, with 49.5% being male participants. The mean weight was around 99.8 ± 18.1 kg, and the average BMI prior to using semaglutide was 36.3 ± 8.3 kg/m^2^, with predominantly overweight and obese individuals (collectively 91.3%). Each comorbidity was diagnosed in around 50.0% of the cohort, with approximately two-thirds (68.6%) of individuals experiencing three to five comorbidities. Diabetes mellitus and dyslipidemia were diagnosed in 48.4% and 48.5% of patients, respectively, highlighting a high burden of metabolic disorders. Hypertension was reported in 48.1% of patients; stroke history was found in 47.8% of individuals; congestive heart failure affected 51.2% of individuals; ischemic heart disease was observed in 50.1% of patients; atrial fibrillation was observed in 50.9% of patients; myocardial infarction was seen in 49.5% of patients; and chronic kidney disease was noted in 48.1% of participants. These findings show that the study population had a high prevalence of cardiovascular and metabolic diseases, making it an appropriate cohort for evaluating the effects of semaglutide. The relatively balanced gender distribution and presence of multiple comorbidities emphasize the clinical relevance of this study, particularly for individuals at risk of cardiometabolic complications. Further details are provided in Table 1.

### 3.2. Clinical Laboratory Testing Monitored Before the Use of Semaglutide

Table 2 presents the laboratory values assessed before initiating semaglutide treatment, providing insight into kidney function, thyroid status, and lipid profile. The average HbA1c was 9 and ranged between 6 and 12, indicating that patients were either prediabetic or had diabetes. Serum creatinine averaged 78.5 ± 19.6 mcmol/L in the study population, indicative of normal renal function. The thyroid function test revealed a mean thyroid-stimulating hormone level of 7.2 ± 3.7 uIU/mI, suggesting possible hypothyroidism. Triiodothyronine and thyroxine provided further context to the assessment of thyroid function, averaging 14.4 ± 1.2 pmoI/L and 15.9 ± 1.1 pmoI/L, respectively. The average for total bilirubin level was 5.8 ± 2.3 umoI/L, suggesting normal hepatic function. The lipid profile results indicated triglyceride levels at 23.8 ± 7.7 mmoI/L, high-density lipoprotein at 1.3 ± 0.4 mmoI/L, low-density lipoprotein at 2.3 ± 1.0 mmoI/L, and total cholesterol at 3.9 ± 1.8 mmoI/L. These values suggest a range of lipid metabolisms, with some individuals potentially having dyslipidemia. These laboratory values served as critical baseline markers for monitoring metabolic, renal, and thyroid-related changes with semaglutide. Despite all aforementioned laboratory tests, pancreatic lipase, amylase, and calcitonin were not measured in all patients.

### 3.3. Disease History Assessment Prior to Semaglutide Utilization

Table 3 shows the frequency with which certain diseases were evaluated in individuals prior to initiating semaglutide. The decision to assess their evaluation was based on the potential occurrence or worsening of parameters with semaglutide. Approximately 48.5% of participants were evaluated for thyroid abnormalities, and 49.0% were checked only for a family history of thyroid cancer. Additionally, 49.9% were asked whether they had experienced pancreatitis in their lives. Regarding neurological and ocular conditions, approximately 52.0% of participants were evaluated for a history of retinopathy, other eye problems, or neuropathy. Despite the necessity of conducting thorough thyroid, pancreatic, and neuronal health assessments prior to using semaglutide, the collective history of these diseases was only checked in 1.8% of individuals, as shown in Table 4. This could predispose the majority to severe consequences, particularly those related to the thyroid, pancreas, and optic nerve. Further details related to the comorbidities assessed prior to using semaglutide are provided in Table 3 and Table 4.

## 4. Discussion

Despite the importance of clinical and disease assessments prior to initiating semaglutide to maintain long-term patient safety, none of the studies in this field have discussed this topic previously. Our findings reveal for the first time a substantial deficiency in pretreatment screening when semaglutide is used off-label for weight reduction [16]. Despite 98.6% of patients undergoing pretreatment evaluations for the risk of at least one serious disease that could be aggravated by semaglutide, only 1.8% undergo screening for all diseases, indicating that the current assessment process falls below standards. This predisposes many patients to a profound risk of thyroid abnormalities or cancer, pancreatitis, retinopathy, and neuropathy, especially if they have a history of such conditions. Without essential baseline tests and disease-pretreatment evaluations, those with abnormal labs, pre-existing disorders, or a family history of these diseases will be at higher risk and might potentially experience serious outcomes.

Based on animal and human studies, several medical conditions warrant careful assessment before semaglutide treatment initiation as pre-existing or currently existing disorders may worsen. A preclinical study demonstrated that semaglutide and other GLP-1 RAs cause a dose-dependent and treatment-duration-dependent increase in thyroid C-cell hyperplasia, adenomas, and medullary thyroid carcinoma (MTC) in rats and mice, which is mediated by the GLP-1 receptors that are abundantly expressed on rodent C-cells [17]. Although the relevance of MTC to humans is uncertain and human epidemiological studies have not confirmed it, GLP-1 RAs are contraindicated in patients with a personal or family history of MTC or MEN 2, according to the prescribing information available from Novo Nordisk [18]. This makes the pretreatment assessment of an individual/family medical history of MTC or MEN 2 mandatory. Conflicting results have been shown in clinical trials and large observational studies regarding the risk of pancreatitis with GLP-1 RAs. Some studies, including meta-analyses, suggest a minimal increase, while others do not [19,20,21,22]. However, reports linking GLP-1 RAs to an increased risk of pancreatitis, specifically in T2DM patients [10,23,24,25] mean that a history of pancreatitis is often considered a precaution or relative contraindication [18]. Although it is imperative to investigate whether a history of pancreatitis is influenced by semaglutide use, research in this area focuses more on the causative association between GLP-1 RAs and pancreatitis in healthy animals [26]. Nevertheless, whether a history of pancreatitis exists or not, the potential risks vs. benefits associated with the use of semaglutide must be assessed before treatment initiation. More importantly, a higher vigilance for symptoms of pancreatitis is warranted once semaglutide is initiated.

Although not assessed in our study, a history of gastroparesis should have also been considered before starting Ozempic^®^. In patients with pre-existing gastroparesis, semaglutide was shown to amplify gastrointestinal paralysis and worsen gastrointestinal symptoms (nausea, vomiting, abdominal pain, and malnutrition) [27]. Therefore, routine GI symptom screening should be advocated, while semaglutide should be avoided in some cases, specifically in high-risk patients. In the SUSTAIN-6 trial, an increase in the risk of diabetic retinopathy (DR) complications was demonstrated (HR 1.76; 95% CI 1.11–2.78), including vitreous hemorrhage and blindness with semaglutide vs. a placebo [5]. This risk was significantly higher in patients with pre-existing retinopathy. Intensive glycemic control with agents such as semaglutide can transiently worsen existing DR during the first 1–2 years [15]. This highlights the need for a baseline retinal exam prior to treatment initiation and close ophthalmologic monitoring in patients with pre-existing DR. Despite the growing body of evidence highlighting serious diseases that are associated with or aggravated by semaglutide, an incomplete assessment was performed in the majority of our subjects; only 1.8% underwent a complete evaluation before starting the treatment.

Current practice lacks standardized protocols to ensure that pretreatment screenings of semaglutide users are followed up and thoroughly performed. These deficiencies include inadequate blood work and incomplete checking of personal and family medical histories. Although conflicting data exist regarding the assessment of certain diseases, such as a history of pancreatitis, and are not weighed heavily enough in the decision to prescribe semaglutide, it is clinically worth checking, given the facts reported in clinical trials. Furthermore, some adverse events, such as gastroparesis, are often underdiagnosed and often mistakenly attributed to “typical” GLP-1 RA side effects, particularly when symptoms such as bloating and vomiting worsen after starting the treatment. Cumulatively, our study highlights the need for validated pretreatment screenings of semaglutide, especially when used for weight management. This procedure must include comprehensive medical and family history checks, specifically for thyroid and pancreatic conditions; baseline and routine laboratory workups covering renal function (serum creatinine and eGFR), hepatic profile (ALT, AST, and bilirubin), pancreatic (lipase and amylase), and thyroid hormones (TSH, T3, and T4); glycemic parameters, including HbA1c checked in non-diabetic individuals to monitor hypoglycemia or unmask latent diabetes; and neurological and ophthalmologic check to assess neuropathy and retinopathy. As media publicity and anecdotal success stories make semaglutide a popular obesity drug, clinicians must avoid abstaining from comprehensive assessments to initiate a quick start. Prescribing semaglutide for weight reduction/management necessitates a cautious approach, unlike lifestyle changes or non-pharmacological interventions. The liberal prescribing of this drug based solely on its benefits may endanger patient safety.

Several limitations exist in our study. First, given its retrospective nature, we relied on the documentation of pretreatment screenings, particularly non-laboratory measures, which could be confirmed with patients verbally; hence, evaluations may have been performed but not recorded. Second, since practices vary by institution and area, this study’s single-center design may limit the generalizability of our results. Third, no inferential statistics were conducted, which limits our understanding of the impact of demographic or clinical variables on the occurrence of adverse events and adds more value to the pretreatment assessment. In other words, the clinical impact of inadequate screening cannot be fully assessed without outcome data, such as adverse event rates or efficacy assessments. Thus, our findings should inspire further research rather than being taken as proof of harm. A prospective, multicenter study is warranted to affirm our findings and quantify adherence to comprehensive screening, including effects on safety and treatment outcomes. Additionally, the testing of screened and unscreened populations could be adopted to compare adverse event rates, weight loss efficacy, and patient satisfaction.

## 5. Conclusions

Pretreatment screening is still underdeveloped in patients who are prescribed semaglutide for weight reduction/management. This could predispose such patients to serious outcomes, particularly in relation to the thyroid, pancreas, and optic nerve.

## Figures and Tables

**Table 1:** Baseline characteristics (n = 715).

Patient Characteristic/Variable	Value
Age [Mean (SD)]	40.2 (12.0)
Gender [Male] (n; %)	354 (49.5)
Weight [Mean (SD)]	99.8 (18.1)
BMI [kg/m^2^] prior to Ozempic^®^ use [Mean (SD)]	36.3 (8.3)
BMI category (n; %)	
Underweight	0 (0.0)
Normal	62 (8.7)
Overweight	114 (15.9)
Type I obesity	310 (43.4)
Type II obesity	229 (32.0)
Comorbidities [Yes] (n; %)	
DM	346 (48.4)
DLD	347 (48.5)
HTN	344 (48.1)
Stroke	342 (47.8)
CHF	366 (51.2)
IHD	358 (50.1)
A-fib	364 (50.9)
MI	354 (49.5)
CKD	344 (48.1)
Number of current comorbidities (n; %)	
0	0 (0.0)
1	13 (1.8)
2	51 (7.1)
3	116 (16.2)
4	202 (28.3)
5	172 (24.1)
6	105 (14.7)
7	46 (6.4)
8	8 (1.1)
9	2 (0.3)

Abbreviations: DM (diabetes mellitus); DLD (dyslipidemia); HTN (hypertension); stroke (cerebrovascular accident); CHF (congestive heart failure); IHD (ischemic heart disease); A-fib (atrial fibrillation); MI (myocardial infarction); CKD (chronic kidney disease).

**Table 2:** Clinical parameters evaluated before using semaglutide.

Clinical Parameter	Value [Mean (SD)]
HbA1C [%]	9.0 (1.7)
Scr [mcmol/L]	78.5 (19.6)
TSH [uIU/mI]	7.2 (3.7)
T3 [pmoI/L]	14.4 (1.2)
T4 [pmoI/L]	15.9 (1.1)
TG [mmoI/L]	23.8 (7.7)
HDL [mmoI/L]	1.3 (0.4)
LDL [mmoI/L]	2.3 (1.0)
Total cholesterol [mmoI/L]	3.9 (1.8)
Total Bilirubin [umoI/L]	5.8 (2.3)
Amylase [U/L]	Not measured
Lipase [U/L]	Not measured
Calcitonin [pg/mL]	Not measured

Abbreviations: HbA1c (glycosylated hemoglobin A1c); Scr (serum creatinine); TSH (thyroid-stimulating hormone); T3 (Triiodothyronine); T4 (thyroxine); TG (triglycerides); HDL (high-density lipoprotein); LDL (low-density lipoprotein).

**Table 3:** Disease history evaluated prior to using semaglutide.

History of Disease	Checking Status	
	Yes (%)	No (%)
History of thyroid abnormality	347 (48.5)	368 (51.5)
Family history of thyroid cancer	350 (49.0)	365 (51.0)
History of pancreatitis	347 (49.9)	358 (50.1)
History of retinopathy	372 (52.0)	343 (48.0)
History of other eye problems	370 (51.7)	345 (48.3)
History of neuropathy	371 (51.9)	344 (48.1)
Any disease [potentially aggravated by semaglutide] evaluated prior to semaglutide administration	705 (98.6)	10 (1.4)

**Table 4:** Patient classification based on the number of diseases evaluated prior to the use of semaglutide.

Number of Diseases [Potentially Aggravated by Ozempic] Evaluated Prior to Semaglutide Administration	Number of Patients Evaluated (%)
0	10 (1.4)
1	74 (10.3)
2	162 (22.7)
3	207 (29.0)
4	175 (24.5)
5	74 (10.3)
6 (all required diseases)	13 (1.8)

## Data Availability

The original contributions presented in this study are included in the article. Further inquiries can be directed to the corresponding author.
